# Beyond the catheter: patient selection and pathways in complex interventional cardiology

**DOI:** 10.1007/s12471-026-02056-4

**Published:** 2026-05-19

**Authors:** Pim van der Harst

**Affiliations:** Department of Cardiology, University Medical Centre Groningen, Groningen, The Netherlands

Three papers in this issue address patient groups outside the standard interventional pathways: spontaneous coronary artery dissection (SCAD), extracorporeal cardiopulmonary resuscitation (ECPR), and transcatheter treatment of pure native aortic regurgitation (AR).

Al-Gully et al. report 117 patients managed within a dedicated SCAD pathway [1]. Conservative management was more frequent, with fewer stents and less dual antiplatelet therapy, without worse outcomes. Recurrence rates were low and similar between groups. The pathway also covers vascular screening, psychosocial support, and SCAD-specific rehabilitation, as important as the interventional choice. Kalkman et al. underlined that SCAD remains under-recognised in a young, female population [2]. Al-Gully and colleagues now show the next step: organising care around it.

Bogerd et al. examined coronary angiography in ECPR patients at Amsterdam UMC [3]. Significant coronary disease was present in 94%, with a culprit lesion in 78% of those reaching the cath lab. In-hospital survival was markedly worse in ST-elevation patients who did not undergo angiography. Ali et al. previously reviewed Dutch ECPR practice, identifying low-flow time and selection as the main outcome determinants [4]. These data extend this into the cath lab: although deferral is often valid, they argue for an early coronary strategy when feasible.

Uchoa de Assis et al. compare the dedicated Trilogy valve (*n* = 21) with off-label transcatheter devices (*n* = 21) for pure native AR [5]. Device success was higher, embolisations were fewer, and residual regurgitation was less with Trilogy. Despite the small, retrospective sample, the result is consistent with what a dedicated valve should achieve without annular calcification.

Three niche cohorts, three different selection problems: whether to intervene (SCAD), which patients still need urgent angiography (ECPR), and matching device to anatomy (AR), each demanding a dedicated approach tailored to the underlying condition.
